# TrichomeYOLO: A Neural Network for Automatic Maize Trichome Counting

**DOI:** 10.34133/plantphenomics.0024

**Published:** 2023-02-28

**Authors:** Jie Xu, Jia Yao, Hang Zhai, Qimeng Li, Qi Xu, Ying Xiang, Yaxi Liu, Tianhong Liu, Huili Ma, Yan Mao, Fengkai Wu, Qingjun Wang, Xuanjun Feng, Jiong Mu, Yanli Lu

**Affiliations:** ^1^ Maize Research Institute, Sichuan Agricultural University, Wenjiang 611130, Sichuan, China.; ^2^ State Key Laboratory of Crop Gene Exploration and Utilization in Southwest China, Sichuan Agricultural University, Wenjiang 611130, Sichuan, China.; ^3^ College of Information Engineering, Sichuan Agricultural University, Yaan 625014, Sichuan, China.; ^4^ Triticeae Research Institute, Sichuan Agricultural University, Wenjiang 611130, Sichuan, China.; ^5^ College of Chemistry and Life Sciences, Chengdu Normal University, Wenjiang 611130, Sichuan, China.

## Abstract

Plant trichomes are epidermal structures with a wide variety of functions in plant development and stress responses. Although the functional importance of trichomes has been realized, the tedious and time-consuming manual phenotyping process greatly limits the research progress of trichome gene cloning. Currently, there are no fully automated methods for identifying maize trichomes. We introduce TrichomeYOLO, an automated trichome counting and measuring method that uses a deep convolutional neural network, to identify the density and length of maize trichomes from scanning electron microscopy images. Our network achieved 92.1% identification accuracy on scanning electron microscopy micrographs of maize leaves, which is much better performed than the other 5 currently mainstream object detection models, Faster R-CNN, YOLOv3, YOLOv5, DETR, and Cascade R-CNN. We applied TrichomeYOLO to investigate trichome variations in a natural population of maize and achieved robust trichome identification. Our method and the pretrained model are open access in Github (https://github.com/yaober/trichomecounter). We believe TrichomeYOLO will help make efficient trichome identification and help facilitate researches on maize trichomes.

## Introduction

Plant trichomes are highly specialized structures with diverse functions in plant development and stress responses. They are outgrowths developed from epidermal pavement cells of different plant tissues, including the leaves, stems, and floral organs. Trichomes can protect plants from insect damage, pathogen attacks, ultraviolet radiation, leaf temperature reduction, and prevent water loss [1–5]. The density and dimensions of the plant trichomes are both important and contributing to these functions. Given the importance of trichomes in defending plants against biotic and abiotic stresses, the major regulators of trichome formation in the model plant, Arabidopsis, have been identified using classical molecular genetic approaches [6]. However, the identification of these genes largely relied on the abundance of mutants in Arabidopsis. The key trichome initiation and patterning regulators are all identified dependent on mutants [7–10]. In contrast to the sophisticated mechanisms of trichome formation that have been revealed in the model eudicot plant, Arabidopsis, the development and function of trichomes in monocots are still largely unknown. In contrast to the abundance of mutants in Arabidopsis, the number of trichome mutants in monocots is far more less, which has limited genetic research such as map-based gene cloning. Although genome-wide association study is widely used in gene identification for agronomical traits, the counting of trichomes manually in thousands of samples is an impractical task for geneticists. For these reasons, only a few genes that directly control trichome formation in monocots have been identified to date. Although researchers have realized the functional importance of trichomes, the tedious and time-consuming manual phenotyping process greatly limits the progress of trichome gene cloning. A high-throughput and accurate phenotyping approach is urgently required to quantify the density and characteristics of plant trichomes.

Owing to the development of computer vision and artificial intelligence, deep learning (DL) has been applied to improve plant trait recognition in recent years [11]. The DL algorithms accelerate and automate image analysis, thereby providing us an opportunity to dissect the complex agronomic traits via a data-driven prediction. A typical framework of computer vision-based image processing consists of 4 parts: preprocessing, feature extraction, neural network, construction and application. The most common preprocessing steps include data transformation and image cropping, which standardize the images and extract the target objects out of them. With feature extraction, a particular organ or featured area can be identified and quantified automatically. Convolutional neural network (CNN)-based approaches have been widely used in plant morphology describing, organ counting, crop postharvest quality assessment [12], plant stress phenotyping [13], and field-based disease classification [14–16]. Additionally, CNN-based approaches have successfully localized plant organs and extracted characteristics in the evaluation of chlorophyll content in cotton leaves [17] and rice root distribution [18]. In maize, a CNN-based approach called TasselNet is proposed to count maize tassels in the field with a relatively high degree of accuracy [19]. Furthermore, a CNN-based northern leaf blight automatic identifying system achieved 96.7% accuracy in maize disease phenotyping. These advances may aid precision molecular breeding for the improvement of disease resistance over a number of plants and disease categories [20].

In addition to the basic CNNs, there are many DL networks that have been built in recent years, including Recurrent Neural Networks [21] and Transformers [22] for sequence prediction, Faster R-CNN [23] and You-Only-Look-Once (YOLO) family for object detection [24,25], and DeepLab for semantic segmentation [26]. A region-based R-CNN model was used for wheat spike detection and attained a relatively high detection accuracy [27]. A deep CNNs (DCNN) was developed for automatic stomata identification and counting in plants [28]. In addition, the Faster R-CNN model was also applied to detect plant stomata movement [29]. DETR (Detection Transformer) is a visual version of Transformer proposed by Facebook AI. It can be used for object detection and panoramic segmentation. This is the first object detection framework to successfully integrate Transformer as the central building block of the detection pipeline. Compared with previous object detection methods, DETR effectively removes the need for many hand-designed components, such as Non-Maximum Suppression and Anchor generation [30]. Cascade R-CNN is a sequential multistage extension that uses the output of the previous stage for the next stage of training. The later the stage, the higher Intersection over Union (IoU) threshold is used to generate a higher-quality box. Cascade R-CNN uses cascade regression as a resampling mechanism to increase the IoU value of the proposal stage by stage, so that the proposals resampled by the previous stage can adapt to the next stage with a higher threshold [31]. Although an increasing number of studies have proposed different DL-based algorithms for describing plant development, no fully automatic method for recognizing maize trichomes have been reported.

Here, we present an automated tool to count and measure maize trichomes from scanning electron microscopy (SEM) images. Our self-developed tool called TrichomeYOLO is open to the community and allows plant researchers to quantify the density and average length of trichomes using our pretrained networks. To construct TrichomeYOLO, we used YOLOv5 as our base model and introduced Transformer into our model to provide hierarchical feature representation and propose a shifted window self-attention approach. Then, we embed bidirectional feature pyramid network (BiFPN) into the model to provide weighted feature fusion mechanism, which helped the model learn the importance of different channels. We applied TrichomeYOLO to assess trichome variations in a natural population of maize and achieved robust trichome identification.

## Materials and Methods

### Plant materials

**Fig. 1.**
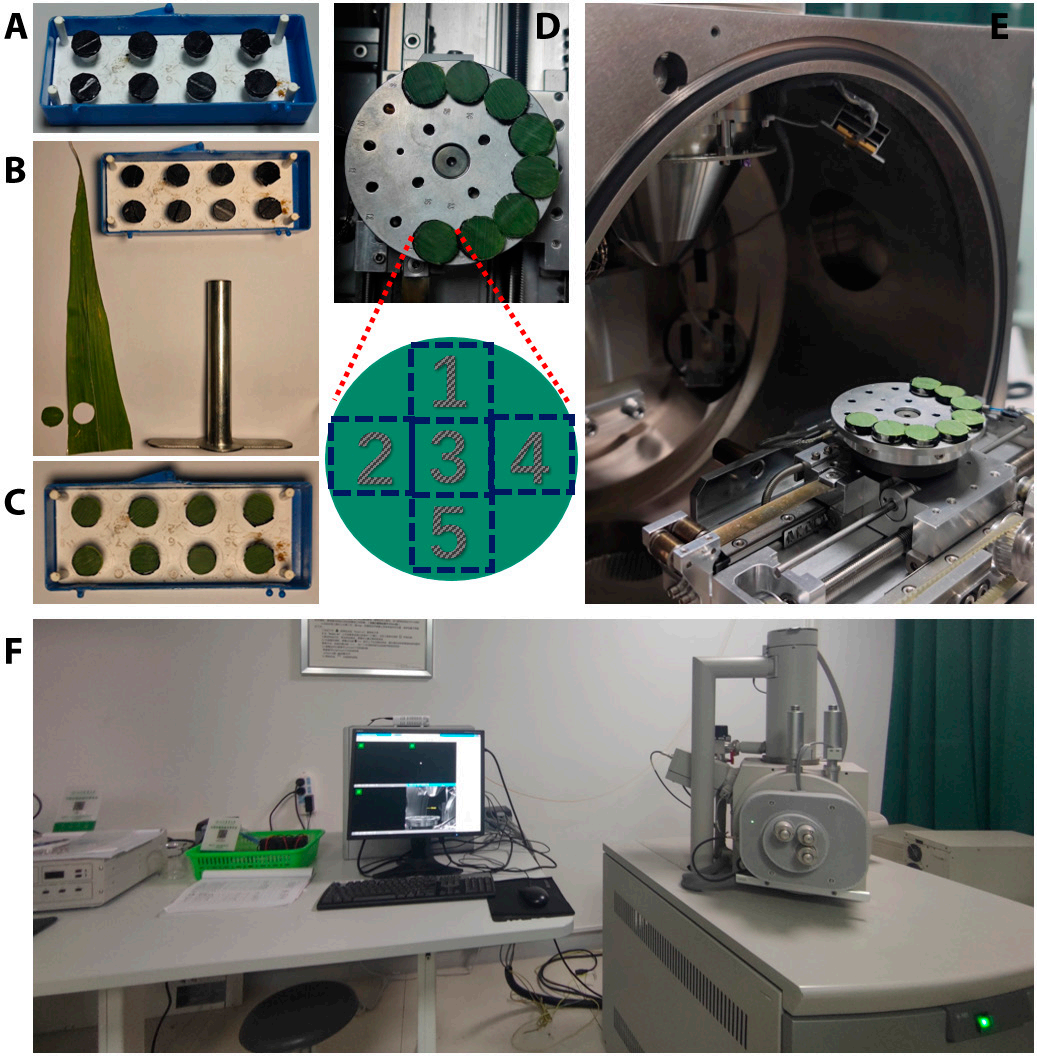
Image acquisition process. (A) Sample holders preparing. (B) About 10 cm from the fresh leaf tips were sampled for microphotography. (C) The samples were placed on sample holders without fixation or sample pretreatment. (D) For each sample, at least 5 fields of view were taken for multifield microphotography. (E) Samples holders were put in the SEM. (F) Ready for image acquisition.

The maize association panel consisted of 370 inbred lines from temperate, tropical, and subtropical areas that were used to scan trichome variations. In the winter of 2020, 3 replications of the above maize inbred lines were planted at the Nanbin Farm of the Chinese Academy of Agricultural Sciences, Sanya, Hainan Province (18°23′47.2″N, 109°12′11.0″E) in a completely randomized design. For each replicate, 5 individuals with consistent growth were chosen, and the leaf above each ear at the silking stage was collected. About 10 cm from the fresh leaf tips were sampled for microphotography, and only the adaxial epidermis of the leaf was scanned for trichome identification. SEM images were obtained using a FEI Quanta 450 FEG SEM system with an acceleration voltage of 15 kV. For each sample, at least 5 fields of view were taken for multifield microphotography to ensure that all the sample information could be covered. The detailed image acquisition process is shown in Fig. 1. The fresh maize leaves were placed on prepared sample holders directly (Fig. 1B) without fixation or sample pretreatment. Thus, the image acquisition rate of the adaxial leaf surface was approximately 1 min per image and 5 min per sample.

### Experimental operation environment

The experimental hardware environment of our study was as follows: CPU: Intel Xeon Gold 5218 CPU; memory: 128G; and graphic card: NVIDIA Tesla V100. All the experiments and analyses are based on Windows Server 2019 equipped with Pytorch 1.11.0, CUDA11.6, CUDNN8.4.0, and Python3.9.12.

### TrichomeYOLO neural network construction

**Fig. 2.**
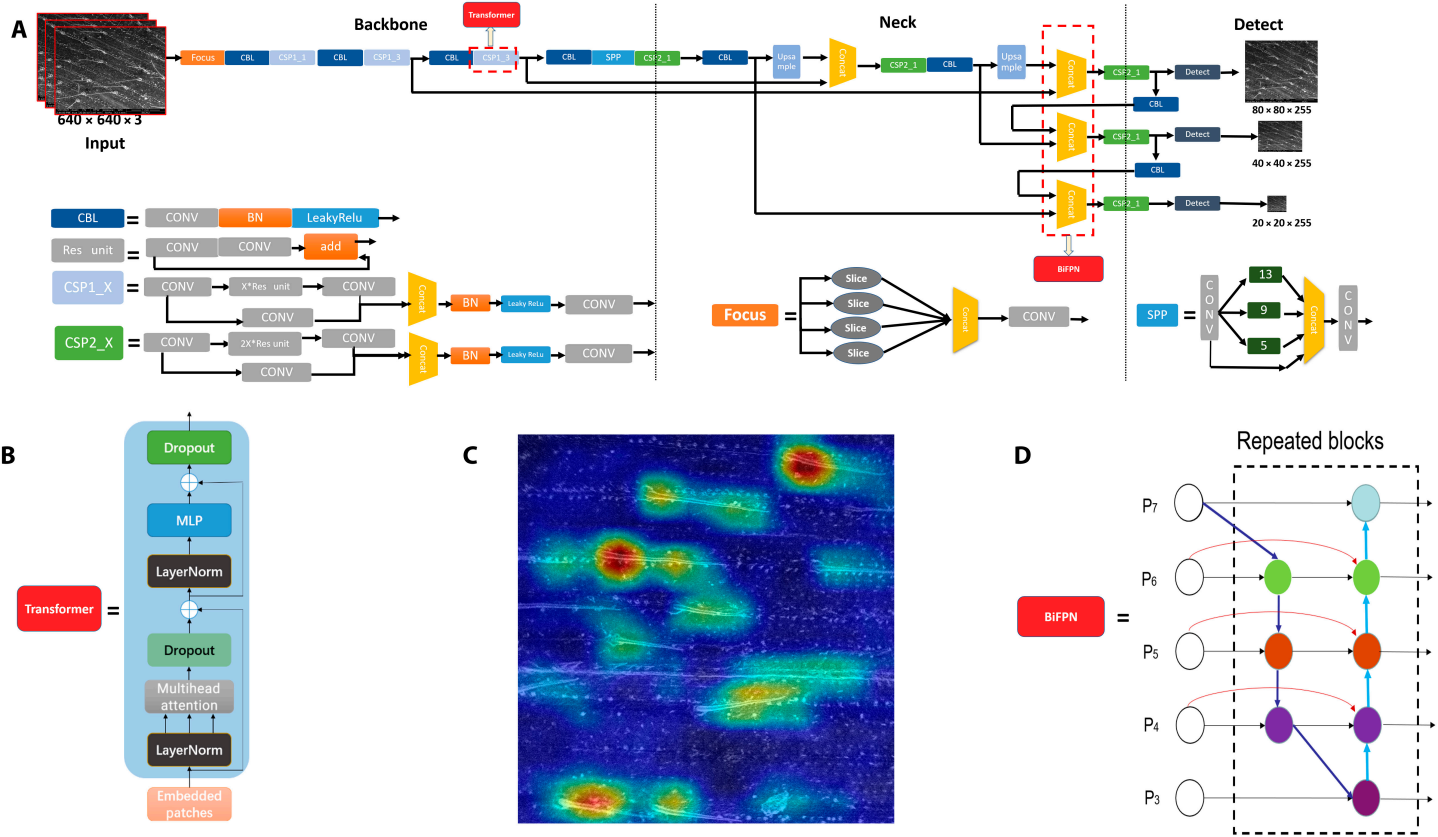
Architecture of the TrichomeYOLO neural network. TrichomeYOLO neural network is constructed on the basis of YOLOv5 with the backbone replaced by Transformer and multiscale feature fusion using weighted bidirectional feature pyramid network (BiFPN). (A) Network architecture of YOLOv5. A patch of trichome images could be input to the detection backbone. The red block indicates the improvement of TrichomeYOLO based on YOLOv5. (B) The structure of original Transformer’s encoder, which has the multihead self-attention. (C) The attention heatmap drawn by TrichomeYOLO. The map highlights the object-specific discriminative regions. (D) The structure of BiFPN, which could enhance the feature extraction ability.

YOLO performs both classification and box regression at the same time, which greatly reduces the time of detection [25]. YOLOv5 [32] is a one-stage detection-based network that consists of an input, backbone, neck, and prediction output, which can realize multilayer feature multiplexing. The backbone includes the Focus structure and the cross-stage partial networks CSPNet. The Focus structure includes 4 slicing operations and 1 convolution operation with 32 convolution kernels, turning the initial 608 × 608 × 3 image into a 304 × 304 × 32 feature map. CSPNet is designed to reach a profound gradient combination while reducing the number of computations by partitioning the feature map of the base layer into 2 parts and then merging them through a proposed cross-stage hierarchy. The neck part contains the path aggregation network (PANet) and spatial pyramid pooling (SPP) modules. The PANet module aggregates high-level feature information with the output features of different layers and then aggregates shallow features through a bottom-up path aggregation structure to fully integrate the image features of different layers. The structure of YOLOv5 is shown in Fig. 2A.

The original YOLOv5 backbone uses Darknet, and we found that, in the case of high-density trichomes, the model has a very high proportion of missed detections. Therefore, we used the Transformer network as the backbone (Fig. 2B).

The Transformer is a classic natural language processing (NLP) model designed by Google’s team in 2017. Because of the computationally efficient nature and scalability of Transformer, it has made outstanding achievements in NLP. Inspired by the success of Transformer extensions in NLP, we attempted to apply standard Transformer directly to images with minimal modifications. The Transformer backbone (Fig. 2B) operates at a constant and relatively high resolution and has a global receptive field at each stage. The great success of Transformer-based models benefits from the powerful multihead self-attention mechanism, which learns token dependencies and encodes contextual information from the input. The core component of a Transformer block is multihead self-attention. The self-attention head is described as [33]:

$$Q_{h}=XW_{h}^{Q},K=XW_{h}^{K},V=XW_{h}^{V}$$

$$A_{h}=\text{softmax}\left(\frac{Q_{h}K_{h}^{T}}{\sqrt{d_{k}}}\right)$$

$$H_{h}=\text{AttentionHead}\left(X\right)=A_{h}V_{h}$$

where *Q*, *K*∈ *R*^*n*×*d_k_*^, *V* ∈ *R*^*n*×*d_v_*^, and the score *A*_*i*,*j*_ indicate how much attention token *x_i_* puts on *x_j_*. There are usually multiple attention heads in a Transformer block. The attention heads follow the same computation despite using different parameters. Let |*h*| denote the number of attention heads in each layer with the output of the multihead attention given by MultiH(*X*) = [*H*_1_, ⋯, *H*_∣*h*∣_]*W^o^*, where *W^o^* ∈ *R*^∣*h* ∣ *d_v_*×*d_x_*^, [·] means concatenation.

**Fig. 3.**
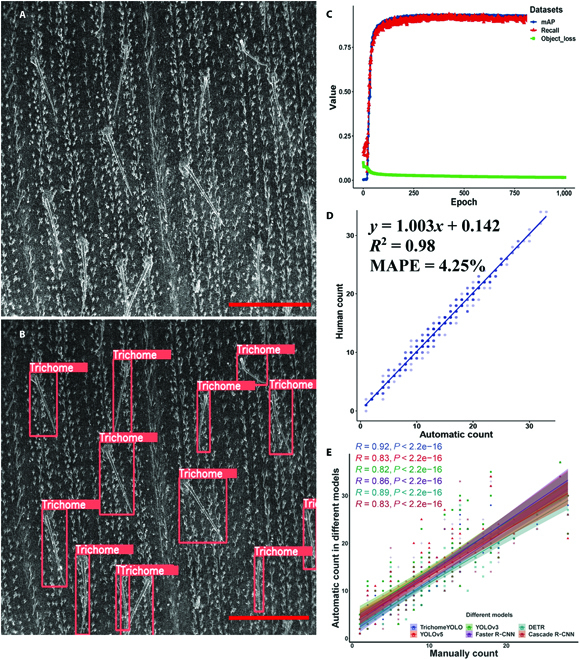
TrichomeYOLO evaluation results. (A) The original image. (B) The trichome detections by TrichomeYOLO. Scale bar, 1 mm. (C) The accuracy of the TrichomeYOLO measured by object loss, mean average precision (mAP), and recall rate (Recall). (D) The scatterplots of the automatically counting against manually counting of the trichome numbers. The shade of the color represents the level of point density. Dark purple indicates higher density of points, and light purple represents lower density of points. (E) The scatterplots of the automatically counting using different models against manually counting of trichome numbers. Different colors represent different models.

These properties enable a dense vision Transformer to provide more fine-grained and globally consistent predictions than fully convolutional networks. From the attention heatmap drawn using Transformer, the attention mechanism in Transformer encoder block effectively gathers the model’s concerns at the base of each trichome (Fig. 2C). By investing more training resources in the main feature areas, the model allows us to effectively improve the accuracy of trichome recognition under high-density conditions.

The original neck layer of YOLOv5 used PANet for cross-scale feature fusion. However, the input features of PANet have different resolutions, and we found that they usually contribute unequally to the fused output features. In addition, it is difficult to identify small objects in target detection. During the convolution process, large objects have more pixels while small objects have fewer pixels. With the deepening of convolution, the characteristics of large objects are much easier to be retained, and the characteristics of small objects are more likely to be ignored. Hence, the feature pyramid network (FPN) was produced for problem solving. In this study, some trichomes, especially in high density, are easy to be ignored. To address this issue, we introduced a simple and efficient weighted BiFPN that incorporates learnable weights to learn the importance of different input features while iteratively applying top-down and bottom-up multiscale feature fusion [34]. Three weighted fusion methods were considered: bagging, boosting, and stacking. BiFPN uses the most efficient fast normalized fusion with the following equation:

$$O=\sum_{i}\frac{\omega_{i}}{\epsilon +\sum_{j}\omega_{j}}*I_{i}$$

where *w_i_* ≥ 0 is guaranteed by applying the Rectified Linear Unit (ReLu) activation function, avoiding numerical instability. Similarly, each normalized weight has a value between 0 and 1, but it is much more efficient because there is no SoftMax operation here. The BiFPN features were fused as follows:

The feature fusion formula:

$$P_{6}^{\mathrm{td}}=\text{Conv}\left(\frac{\omega_{1}*P_{6}^{\text{in}}+\omega_{2}*\text{Resize}\left(P_{7}^{\text{in}}\right)}{\omega_{1}+\omega_{2}+\epsilon }\right)$$

$$P_{6}^{\text{out}}=\text{Conv}\left(\frac{\omega_{1}^{\prime }*P_{6}^{\text{in}}+\omega_{2}^{\prime }*P_{6}^{\mathrm{td}}+\omega_{3}^{\prime }*\text{Resize}\left(P_{5}^{\text{out}}\right)}{\omega_{1}^{\prime }+\omega_{2}^{\prime }+\omega_{3}^{\prime }+\epsilon }\right)$$

where $P_{6}^{\mathrm{td}}\;$ is the intermediate feature of layer 6 in the top-down path, and $P_{6}^{\text{out}}$ is the output feature of layer 6 in the bottom-up path. All the other features were constructed in a similar manner.

Therefore, we formally present TrichomeYOLO, an algorithm that integrates Transformer and BiFPN and achieves a self-organizing tree algorithm. The improved structure is shown in Fig. 2.

### Comparisons of different algorithms in trichome identification

To assess the performance of different algorithms in trichome identification, we introduced the mainstream object detection algorithms YOLOv3, YOLOv5, Faster R-CNN, DETR, and Cascade R-CNN for comparison. YOLOv3 is the most typical version of the YOLO series, and Darknet-53 is the backbone of YOLOv3. Darknet-53 mainly includes 2 network layers: convolutional layer and residual block. In each convolutional layer, after completion of the convolution operation with various convolution kernels, batch normalization is performed, and the LeakyReLU function is then used for activation. Compared with R-CNN, Faster R-CNN has higher accuracy of target detection, and its training process adopts a multitask loss function, and can update the parameters of all network layers without additional disk space to store features. DETR is a visual version of Transformer proposed by Facebook AI, and can be used for object detection and panoramic segmentation [30]. Compared with previous object detection methods, DETR effectively removes the need for hand-designed components, such as Non-Maximum Suppression, Anchor generation, etc. Cascade R-CNN is a sequential multistage extension that uses cascade regression as a resampling mechanism to increase the IoU value of the proposal stage by stage, so that the proposals resampled by the previous stage can adapt to the next stage with a higher threshold [31]. The later the stage, the higher the IoU threshold required to generate a higher-quality box.

### Estimate the length of each trichome

According to the detected picture, we removed the end point coordinates of the detection frame and calculated the diagonal length using the following formula:

$$v=\sqrt[{2}]{\left(x_{1}-x_{2}\right)+{\left(y_{1}-y_{2}\right)}^{2}}$$

where *x*_1_ and *x*_2_ represent the abscissas of coordinates 1 and 2, respectively, and *y*_1_ and *y*_2_ represent the ordinates of coordinates 1 and 2, respectively. The estimated trichome length was then corrected using the length of the bar in each figure. The majority of the measured trichome length or the median of the measured length could be centered near the true value of trichome length. Therefore, we removed the outliers to eliminate the impact of different views and the curling of the trichome, which confirms the average length of trichomes in each image that could be representative. The standard we applied here was as follows: the value >Q3 + 1.5 IQR (interquartile range) or <Q1 − 1.5 IQR (Q1 and Q3 are the first quantile and third quantile of the data, respectively. IQR = Q3 − Q1).

### Statistical analyses

Identification accuracy, *A*, is defined as:

$$A=\frac{\mathrm{TP}+\mathrm{TN}}{\mathrm{TP}+\mathrm{TN}+\mathrm{FP}+\mathrm{TP}}$$

where TP, TN, FP, and FN indicate the number of true positives, true negatives, false positives, and false negatives, respectively. The number of trichomes in each image that were counted manually was assumed to contain only true positives, and the number generated by automatic recognition was assumed to contain both true and false positives. Thus, the precision and recall rates are defined as follows:

$$\text{Precision}=\log\left(\frac{\text{manually counting number}}{\text{automatic counting number}}\right)$$

$$\text{Recall}=\frac{\mathrm{TP}}{\mathrm{TP}+\mathrm{FN}}$$

Linear regression was performed to reveal the relationship between the number of trichomes detected manually and automatically using R (version 3.6.2).

## Results

### Identification accuracy of TrichomeYOLO

A total of 9,614 SEM scanning images were collected from a natural maize population consisting of 370 inbred maize lines from temperate, tropical, and subtropical areas. A total of 1,000 images were randomly selected and split into training (70%; 700 images) and validation (30%; 300 images) groups.

In our model, the TrichomeYOLO algorithm was applied for trichome detection, which integrates Transformer and BiFPN into YOLOv5 and unites image pyramid module and multiscale training into a multiscale feature detection. The model was trained using 700 maize leaf trichome images. The detection results are shown in Fig. 3B. The remaining 300 images were used for model validation, and its performance was evaluated using the object loss rate, mean average precision (mAP), and recall rate (Recall). mAP shows how well the model detects objects, and Recall defines the fraction of retrieved instances among all relevant instances. The loss curve for the algorithm is shown in Fig. 3C. With an increase in the epoch number, the object loss rate decreased significantly, and the mAP of the prediction model increased significantly. The average precision and recall rates were 92.1% and 93.2%, respectively (Fig. 3C). We also compared the number of trichomes measured from maize leaves by humans and by automatically counting in another 500 randomly selected images. The square of the correlation coefficient (*R*^2^) and the mean absolute percentage error (MAPE) between automatically counting using TrichomeYOLO and manually counting were 0.98% and 4.25%, respectively (Fig. 3D).

**Fig. 4.**
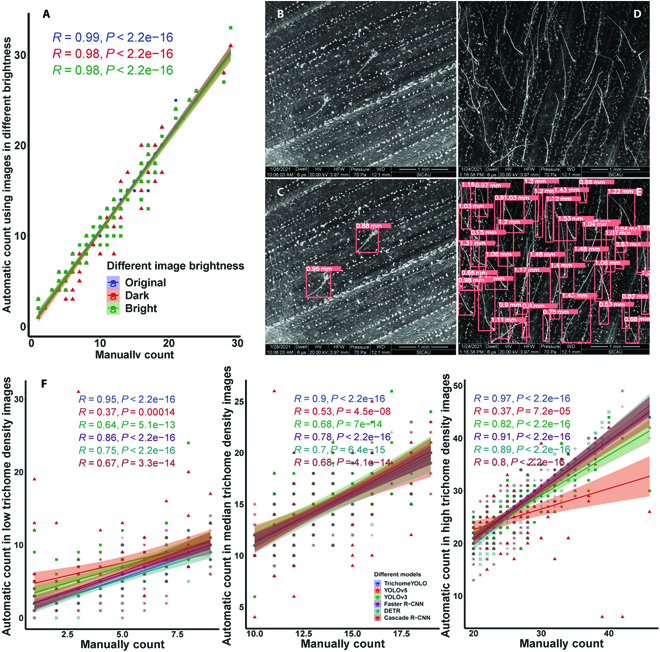
Robust performance of TrichomeYOLO. (A) The scatterplots of the automatic count using TrichomeYOLO against manual count of trichome numbers using the same images with different brightness. Original: The brightness of the input image is normal. Bright: Manually double the brightness of the image. Dark: Manually reduce the brightness of the image to half. The original images with low-density (B) or high-density (D) trichome. The trichome detection results by TrichomeYOLO with low (C) or high (E) density. (F) The scatterplots of the automatic count using different models against manual count of trichome numbers. The Pearson correlation coefficients and *P* values of each model are in different colors.

To further evaluate the performance of TrichomeYOLO, 5 presently prevailing object detection models, Faster R-CNN, YOLOv3, YOLOv5, DETR, and Cascade R-CNN were used to identify trichomes in 100 randomly selected images from the unknown dataset and compare the corresponding detection results. Each algorithm was trained on the same image dataset. The indicators of different models, including the image input size, mAP, processing speed, and parameters, are shown in Table. The TrichomeYOLO model provides the highest accuracy among 6 models when detecting on the same images (with equal input size), which reached a detection accuracy of 92.1%. Because the Transformer and BiFPN modules are added into TrichomeYOLO, the processing speed of TrichomeYOLO is slower than the original YOLOv3 and YOLOv5. Although the speed of TrichomeYOLO and Faster R-CNN are the same, the mAP of TrichomeYOLO is higher than Faster R-CNN. Benefiting from the basic structure of YOLOv5, the parameters of our model is much lighter than that of the other 2-stage algorithms, DETR and Cascade R-CNN (Table). To further estimate the accuracy of those algorithms, the Pearson correlation coefficient (*R*) of the automatically counting and manually counting in TrichomeYOLO, YOLOv5, YOLOv3, Faster R-CNN, DETR, and Cascade R-CNN were 0.92, 0.83, 0.82, 0.86, 0.89, and 0.83, respectively (Fig. 3E), indicating the advantages of Transformer and BiFPN in TrichomeYOLO.

**Table.** Comparisons between the TrichomeYOLO and 5 mainstream algorithms.

Method	TrichomeYOLO	YOLOv5	YOLOv3	Faster R-CNN	DETR	Cascade R-CNN
Input size	640 × 640 × 3	640 × 640 × 3	640 × 640 × 3	640 × 640 × 3	640 × 640 × 3	640 × 640 × 3
mAP@0.5 (%)	92.1	89.3	86.3	89.4	89.0	81.0
Speed (s)	0.5	0.1	0.1	0.5	0.8	0.7
# Params (M)	7.2	7.1	61.4	41.12	36.74	44.3

In general, the brightness of images can affect the accuracy of detection. The brightness of images collected for different samples are different, and manual brightness control is not feasible, which requires the model to perform insensitively to brightness. To test the robustness of TrichomeYOLO, randomly selected 100 images with manually double and half the brightness of the image to test the model detection accuracy. The Pearson correlation coefficients of the real trichome number and automatically counted number using images under different brightness levels are shown in Fig. 4A. The overbright and dark images both slightly reduced the correlation between the real number of trichomes and the model predicted values (Fig. 4A), indicating that the brightness of the images had a limited impact on TrichomeYOLO’s trichome detection accuracy. In addition, for different trichome densities (Fig. 4B to E), the Pearson correlation coefficients of the manually counted trichome numbers and TrichomeYOLO automatically counted numbers were 0.97, 0.90, and 0.95 for high-density (trichome number in each image: 20 to 46), medium-density (trichome number in each image: 10 to 19), and low-density (trichome number in each image: 1 to 9) trichome counting, respectively (Fig. 4F). In addition, the performance of the other 5 algorithms is also evaluated using the same images. TrichomeYOLO shows stable and high accuracy across different trichome densities. Similar to TrichomeYOLO, the 2-stage algorithms, Fast R-CNN and Cascade R-CNN, both achieve a relative higher accuracy in images with high-density trichome with Pearson correlation coefficients of 0.91 and 0.89, respectively. The original version of YOLOv5 and YOLOv3 perform both poorly, especially when applied to images with low- or high-trichome density (Fig. 4F).

### Measurement accuracy of trichome length

**Fig. 5.**
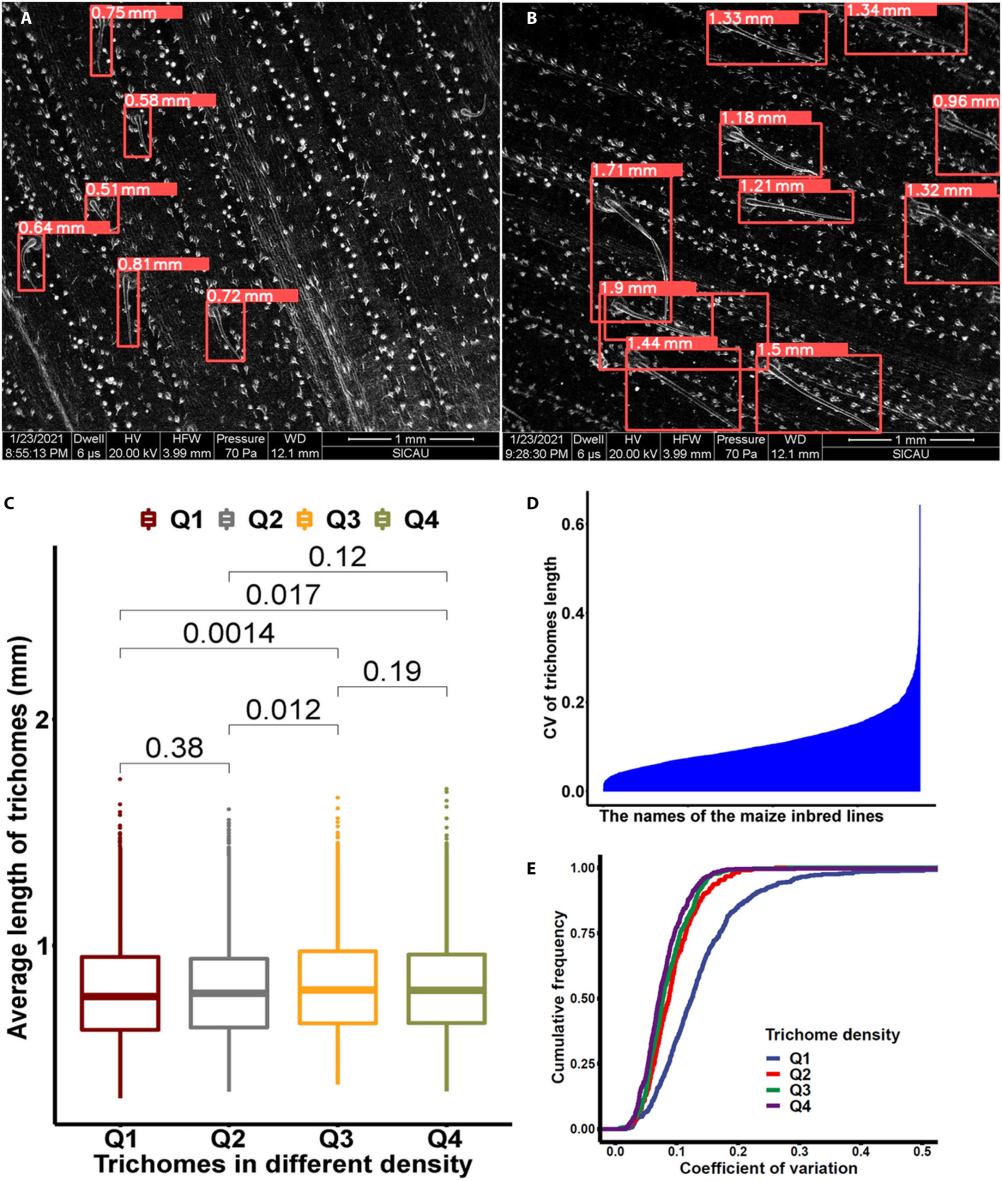
Results of maize trichome length. Short (A) and long (B) trichomes detected in different samples. (C) The boxplot of trichome length with different trichome density. Q1 to Q4 represent the first to last quantile of the trichome density. The numbers between 2 groups are the *P* values of Student’s *t* test. (D) The coefficient of variation (CV) among different samples. (E) The cumulative frequencies of the CV for trichome length. Q1 to Q4 represent the first to last quantile of the trichome density.

The end point coordinates of the detection frame were used to calculate the diagonal length for the measurement of each trichome identified. Moreover, the diagonal length was then divided by the bar length to get the relative length of the trichome to the bar (1 mm) in each image. Because most of the trichomes in each image are in the same condition and only limited number of trichomes were curled or skewed, we removed the outliers to eliminate the impact of different views and trichome curling, which confirms that the average length of trichomes in each image could be representative. Additionally, in our experimental design, each sample was selected for multifield microphotography. Hence, the average length of trichomes in images from the same sample should theoretically be the same. Thus, the variance in trichome length estimated for each sample is an effective evaluation indicator for length detection accuracy. The lengths of the trichomes varied among the different samples (Fig. 5A and B). The difference in trichome lengths at various densities is shown in Fig. 5C. The average length of trichomes with low density (Q1: in the bottom quantile of trichome number) was 0.803 mm, which is slightly but significantly shorter than that of those with high density (0.819 mm; Q4: in the top quantile of trichome number; Student’s *t* test, *P* = 0.017). The coefficient of variation (CV) among different samples varied, ranging from 0 to 0.642, with an average of 0.112. The CV of 95% of the samples we tested is less than 0.216 (Fig. 5D). The lower CV means that the length measuring from the same maize inbred line is quite stable, indicating the good representativity of our method. In addition, we noticed that the CV of trichome length in the Q1 group (low trichome density) was much larger than others with higher trichome density when we analyzed the cumulative frequencies of the CV for trichome length (Fig. 5E).

## Discussion

Plant trichomes are important but easily ignored functional traits. Decades of research have revealed that trichomes can help plants defend themselves against both biotic and abiotic stresses [1–5]. However, the formations and function of trichomes in plants remain largely unknown because of difficulties in trichome counting and measurement. Manual phenotyping of trichome counts is a laborious task with a high risk of errors, let alone measurement of trichome length. In this study, we introduce TrichomeYOLO, a high-precision automated maize trichome counting and measuring method that uses a DCNN to identify the density and length of maize trichomes from SEM images. For plant trichome identifications, there are 2 machine vision methods proposed to identify the trichomes in soybean and Arabidopsis [35,36]. Unfortunately, neither of these models could be used for maize trichome identification. The algorithm developed by Mirnezami et al. [36] focused on image processing with human intervention, with an accuracy close to 90%. Garcia et al. [35] used machine learning to perform trichome identification in Arabidopsis and found a higher variability and difficulty of the analysis as the densities of trichome increase. Actually, trichome overlapping events were also detected numerously and brought inaccuracy to trichome identification in maize. Our contribution introduces a DL-based method for maize trichome identification. In addition, we embed transformer and BiFPN in our model to increase the accuracy when trichomes are in high density, which may serve as an example to solve the trichome crossing-over problem in the other plants. Our method is the first to automatically identify and count maize trichomes using the world’s leading target detection algorithm that combines the Transformer and BiFPN models (Fig. 2). The self-attention module Transformer allows us to maintain a very accurate recognition rate even at very high trichome densities, while BiFPN is a more efficient multiscale fusion method that retains richer information. Five current mainstream object detection models, Faster R-CNN, YOLOv3, YOLOv5, DETR, and Cascade R-CNN, were also used to identify trichomes and compare the associated performance with TrichomeYOLO. Our model provides the highest accuracy at an acceptable speed among 6 models when detecting trichomes on the same images. Although TrichomeYOLO is not the fastest detection model, we believe that it gives us the best balance of speed and accuracy and satisfy the needs of real-time detecting. To test the robust performance of TrichomeYOLO, different images from the unknown data (not the training or testing dataset) were randomly selected and the accuracy of TrichomeYOLO on the unknown dataset was a better performance than the training/testing datasets as shown in Figs. 3D and E and 4A. In addition, our model still has good performance in an extremely bright situation. In general, the image brightness could be solved in the data augmentation section. However, the brightness of images collected for different samples is different. Hence, manual brightness control is not feasible, which requires the model to perform insensitively to brightness. In some situations, manual brightness control could bring a noisy background. We aim to develop a tool, which could be used in different laboratories with images collected in various brightnesses, and TrichomeYOLO could perfectly deal with the brightness issue. In addition, we tested the robustness of TrichomeYOLO using extremely bright, dark, or high trichome density images. It outperformed mainstream target detection models in various conditions, suggesting that TrichomeYOLO is the optimal solution for maize trichome identification at this stage. We found the Pearson correlation coefficients of the manually count trichome number, and TrichomeYOLO automatically count for high-density trichome counting that is higher than those lower-density ones. There are 2 main reasons for this result. On the one hand, Transformer and BiFPN are chosen to solve the problem brought on by high-density trichomes, which could improve the performance of model on high-density trichome counting. On the other hand, there are 3 different types of trichomes on maize leaves. They are macrohairs, prickle hairs, and bicellular microhairs [37]. The macrohairs are the prominent and the most obvious target. We found the less number of trichomes (macrohairs) accompanied by the stronger of prickle hairs as shown in the following figure, leading to inaccurate identification.

Accurate plant phenotyping is important in gaining a fundamental understanding of phenotype–genotype–environment interactions and is critical for plant breeding and agricultural precision management. Computer vision methods, represented by target detection, use an image and computer to make a machine “see.” With the development of computer vision, DCNN is applied in a growing number of phenotyping studies in biological research. In our TrichomeYOLO, the high degree of precision and the extraordinary convergence speed are ideal for trichome detection. Our results demonstrate that the methods developed can reliably quantify the number and length of maize leaf trichomes. We must admit that our model may become obsolete as technology iterates in the future; however, at least now, TrichomeYOLO is the first tool for maize trichome phenotyping. In addition, we believe that the contribution of this study is not only TrichomeYOLO but also to provide breeders with a new and improved phenotyping method for long-term ignored traits. We will provide all the images we collected and the corresponding manual annotations to facilitate research on plant trichomes. We welcome images of different species, from our colleagues worldwide, which will help enhance the detection range and accuracy of TrichomeYOLO.

## Data Availability

The training and test set micrographs, as well as the TrichomeYOLO model, are available on Github (https://github.com/yaober/trichomecounter).
